# Hysteria: Report of a Strange Case

**Published:** 1891-09

**Authors:** John Pope Stewart

**Affiliations:** Attalla, Ala.; Counsellor Elect of the Medical Association, State of Alabama


### HYSTERIA: REPORT OF A STRANGE CASE.*

BY JOHN POPE STEWART, M. D., ATTALLA, ALA.
Counsellor Elect of the Medical Association, State of Alabama.

I have selected for your entertainment a disease called hysteria. I do not mean as it is understood by the laity, or even as it is understood among some of the profession; neither do I mean hypochondriasis, or any trouble speculative, imaginary or conjured.

But I propose to entertain you with the description of a disease most wonderful in its nature, most obscure in its etiology, most marked in its symptoms and one that has as yet remained a mystery to the etiologist, the microscopist and the pathologist. And while calling your attention to this, I shall describe one of the strangest cases I ever saw or ever read of.

The bacillus of hysteria is an undiscovered proto-germ, the pathology an unobserved abnormality; *post mortem* investigation has given no clew to the morbid process. And yet, hysteria is a true and legitimate disease, most difficult to manage and most unsatisfactory to treat.

Hysteria is a disease of the nervous system; that every one will admit, but what part of the nervous system? The brain, the spine, the sympathetic? All, or a few, one or more? If it affects the brain, in what way does it affect the brain? Like cold, like heat, like compression, like concussions, like inflammation, like congestion, like depletion, like paralysis, like anæsthesia? Wholly or in part, or what? Echo answers, "What?"

The fool has hysteria, the most intelligent, the most illiterate or the best informed. A functional nervous disease without organic lesion, without a pathology, without morbidity, without a microsity; and yet, gentlemen, a disease, and should be called by its name, and those who are suffering from it should be informed of their trouble intelligently, instructively and minutely;

*Read before Alabama State Medical Association.

so that they will understand that on their part it is an uncontrollable trouble, and that they are not suspected of sham.

Hysteria, as I said before, gives nothing to morbid anatomy, the brain, the spinal cord, the sympathetic nerve gives no evidence as to its having been present. Hysteria rarely causes death, but hysterical patients dying of intercurrent affections have been examined after death most searchingly for some evidence of this disease, yet nothing has been found. Looking at the brain as a complex organ from which is evolved a conglomerate force called the mind, we can easily appreciate disorders of its functions that are not absolute lesions, the effect of which might be excess or diminution in quality, tone or action. The mind, as we know, is made up of several sub-forces, such as the perception, the intellect, the emotions, the will, etc., and when any one of these become disordered, we have those degrees of insanity which follow each several sub-force diseased. Well, hysteria, we are compelled to admit is purely a physical trouble, and essentially consists in the predominance of the emotions over the will, and to a certain degree the intellect. An example familiar to you all, of the influence of emotion over the will, is demonstrated in the emotion of fear; it renders the sensibility more acute and produces trembling or clonic spasm; again, in brief we have tonic contractions. In surprise or horror we get paralysis of muscular action, joy or anger destroys sensibility to pain, etc. These examples show how the emotions can predominate over every other sub-force of the strongest mind for a short duration of time. So in hysteria, during the exaltation of the emotional power, the power of the will is not only relatively (like in fear, joy, etc.) but absolutely diminished, and these two factors, acting together, the one exalted, the other diminished, continuously, persistently and steadily, produce many of the manifestations of this disease.

The spinal cord, no doubt, and its accessories is secondarily affected, for through the spinal nerves, you know, we have the variety of convulsions, spasms, contractions and the paraplegic and hemiplegic phenomena found in this as well as other diseases.

To detail a differential diagnosis of hysteria with other diseases would take volumes. The physician must be his own judge, for almost every known disease is simulated, and it takes no end of trouble sometimes to tell the true from the false. The hysterical patient tries with utmost and consummate skill to impress others with the belief that they are very, very sick, even unto death. They will try to arouse your sympathy, and failing in that, your fears, and leave nothing undone that will lead you to a belief in their dangerous condition. But careful watching and thorough skepticism on the physician's part, and his diagnosis will be easy. But after you have determined that the trouble is hysteria, you have at last a disease and one that is extremely difficult to manage, so you have, in jumping out of the frying-pan, got into the fire.

Hysteria appears commonly among females. I say commonly, for I have observed many instances where the opposite sex has suffered with this trouble. It attacks the old and young alike, and even has been noticed in infancy. However, it most frequently appears between the ages of ten to thirty years. The most constant and important, and, I may say, consistent factor in the etiology is heredity. This has been observed more than any other point in its history, yet this fails in as many cases as it proves true, and unless every little nervous foible or mental peculiarity can come under the head of hysteria, this supposed heredity is a fallacy.

Other supposed causes for hysteria are almost as groundless. I had some of the worst of cases to which I could attribute no cause whatever, yet it would be well to append just here a list of some of the causes predisposing hysteria, viz. : Ill-directed training of the mind, depressing influences, unhappy surroundings, desires ungratified, continued anxiety, the advent of other diseases, especially diseases of the reproductive organs, menstruation, pregnancy, severe trauma, shocks, physical or mental.

One of the most remarkable cases, however, I ever had, came upon a young lady at the supper table. She, with the family, was partaking of the evening meal, and without any warning or any shock of any description, without any trouble of any nature to which I could attribute a cause, she was seized with a hysterical fit; screamed and laughed and cried alternately; shivered with cold one minute and burned with fever another; was dying this minute, and again felt well and perfectly happy the next; hated everybody this instant, the next loved the whole world; and, in fact, a psychical phenomenon and a symptomatic puzzle. This patient, after six or eight of these attacks, has, under treatment, recovered, and bids fair never to have any more.

The prognosis of hysteria from a single paroxysm of any kind is of course favorable, provided proper treatment is employed, but as to liability to a return of further attacks, much depends on circumstances, the surroundings of the patient, and the time that they have been subject to the trouble. All these have to be taken into consideration.

Under unfavorable circumstances, I wouldn't advise any physician to take charge of a case of this nature, unless there was plenty of money in it, for that would be the only satisfaction he would ever get out of it. For hysteria is purely an emotional disease, and any unfavorable or disagreeable surroundings will set this sub-force into wonderful activity, and your patient is like a roaring maniac, or has gone off into a trance, or has taken on violent convulsions. In fact, a storm has suddenly come up when you thought all was fair weather.

The most remarkable manifestation of hysteria is the epileptiform. This is, I presume, a rare trouble, and is called hystero-epilepsy. I only had one case, and I think it a most extraordinary one, equaling fully the cases related by Charcot and M. Bournville of Madame Leo and Mademoiselle Louise Lateau.

On the morning of January 5th last I was called to see a lady whom I will call Miss M. I walked into the room and found her sitting up in the bed, her forehead pressed forward against her knees, her arms drawn round till the backs of her hands rested against her sides. Her feet were curved over and under. Her face was contorted all out of shape. Thus she was, moaning distressingly, her friends standing around her crying and taking on like they thought she was going to die every minute. I had never seen her before, therefore couldn't tell what the trouble was. I gave her a large dose of morphine hypodermically and chloroformed her, and while she was relaxed and resting under this treatment I took my bearings, conferred with the family, and this is the result :

Miss M. is a maid, 24 years old, black curly hair, large brown eyes, arching black brows, beautiful even, white teeth, large mouth, red lips, form spare made yet symmetrical, skin dark, muddy, goosefleshed and scaly. Her history, as I have gathered from time to time, is that she has been subject to these attacks for four years. She has been treated by five or six doctors, and has gone through the whole *materia medica* in the way of physic, and has had uterine treatment of various kinds. She was wearing a stem pessary for prolapsus at this time.

Her mother is hysterical, of the pretending kind, a virago and a shrew. Her father is a simple-minded rustic. She has a brother in the Alabama Insane Hospital, and another that ought to be in the coal mines. Miss M. herself is fairly intelligent, quick to understand and a bright talker.

The next day the attack returned and I had a good opportunity to see her through it from beginning to end. She began with a slight trembling in her hands, then her arms, then her shoulders, head, neck, legs, until at last the whole body was shaking as with an ague. This grew more and more distinct until it resembled clonic spasms as in epilepsy. This lasted about an hour, then suddenly the spasms became tonic and the body would be drawn up in the tetanus form until only the back of the head and heels would touch the bed. This lasted two or three minutes, and then she dropped back to again slowly rise in the same position. Repeating this some five or six times, she suddenly changed into clonic spasms again, mostly with the head shoulders and arms, which began *poco* at first, rapidly getting *allegro* and finally quite *fortissimo*, her teeth chattering together and her eye snapping rapidly. This lasted about twenty minutes, then a tonic spasm of the muscles of the neck followed, drawing her head back until it seemed to lie between the shoulder blades. Her hands and arms were drawn over and under, the pectoral muscles seemed affected and breathing became quite difficult.

She moaned and gasped for breath. This lasted ten minutes, then again rapid clonic spasms took place, jerking her head about like a bell clapper. This anon ceased, and she shut up like a knife, her head being drawn down to her knees, and if we tried to lay her down her legs would follow. Again the tonic gave way to clonic spasms, and clonic to tonic. Sometimes she would be drawn laterally, sometimes forward, sometimes backward. A hand alone would be affected, again a foot, a leg, an arm, the neck, the back, the abdominal muscles, and again the whole body. She would assume various positions. I have placed her feet in a chair and head on the bed when she would be in the tetani-form condition, and she would hold up from two to five minutes. Another peculiar motion she had was a spasmodic beating of her buttocks rapidly against the bed while on her back, such as Hammond calls hysteria libidinosa. Sometimes she would get perfectly still, nothing moving except her eyes, her head thrown far back, and seemingly hunt for something with her glance over the headboard, giving a grunting moan the while, and now and then jump as if she had discovered what she sought and that it frightened her. She is conscious most of the time during an attack, and will constantly call your attention to the manner in which her trouble is affecting her. There are moments, however, that she seems to know nothing. She has great difficulty in swallowing and is often strangled with a little water. Her mouth gets as dry as a bone. Her breath is dry, hot and offensive. She sweats like a horse when she is working through one of these spells. She often chews "at" her tongue, but never wounds it. She grinds her teeth so hard and loud that it makes one's flesh creep to hear her.

She twice has assumed the cruciform position and became cataleptic. In her most rigid state she is perfectly conscious and makes plaint of the pain that accompanies each spasm and calls them cramps.

She has great difficulty in making water, which is scanty and high colored. Her bowels are usually costive. Her menses are irregular and lacking in qauntity and quality.

Her appetite is usually good, even just after an attack. She is very sore a day or two following a spell, and is unable to walk on that account and weakness together. She has a kind of rheumatism in her left side, hip and legs. There is no swelling or other symptom to locate it. She feels it, nevertheless, she says.

There is no perceptible change in the heart's action. Throughout one of the attacks it remains strong and steady and beats away with normal regularity.

There is no change in the pupil of the eye; neither contraction nor dilatation of the slightest degree can be detected at any period of the attack.

She has an attack about once a week. Sometimes, however, she may have four or five during a week. She is not particular as to date or time of day or night, however. She has not gone longer than a week for the last eight or ten months. Their duration is never the same. They may begin in the afternoon and run on all through the night, to rest a few hours, to start up again and last another ten to sixteen hours; or, again, they may not last over an hour or so; and, of course, they do not run through her whole programme at each engagement.

One peculiarity I have noticed, and by it have been led to believe that there was some method in her madness. If there is a large crowd of friends or acquaintances around her bedside, to sympathize with her and to make exclamations of wonder and astonishment, and especially if there happens to be a new face present, she, it seems, goes through her whole performance for their benefit.

She suffers greatly with headaches all the time during the intervals as well as the attacks. She has a continual pain in the left ovarian region, of a dull nature, during the intervals, that grows very acute during an attack. Her stomach is a great source of trouble, and seems always deranged. She vomits often during the paroxysm and especially immediately afterwards.

During the intermissions of these phenomenal periods her whole talk and conversation is concerning her condition. Her whole time is taken up by relating to new acquaintances the history of her trouble and its many peculiar manifestations. She has no aim or object in life. She doesn't try to do anything, only surprise her family and physician by developing some new feature into her seance.

My treatment has been along the usual line of remedies for hysteria, such as moral suasion, reasoning, kindness, sympathy, sarcasm, acerbity, anger, terror. Everything in that line failed. I gave her the bromides of potassium, calcium, sodium and zinc, and then mono-bromate of camphor, also electricity, strychnia, phosphorus and iron. All failed. The paroxysms would return with just as much force and last just as long. It looked like pouring water into a sinkhole for all the good that my treatment had done. I also employed other remedies for her uterine troubles and for the irregularity of the catamenia. I believe that I have omitted to mention that she was troubled with an irregularity of this nature and that she would go from five to six weeks sometimes, and during the menstrual period her hystero-epileptic paroxysm would be the most violent.

And now for the sequel, three weeks ago I ordered from Birmingham 15 grains of the chloride of gold and sodium. I gave her the first dose the 1st day of April. Since then she hasn't had a single symptom of her trouble. I'm giving her the 20th of a grain of the double chloride three times a day in an aqueous solution. Last Thursday, the 9th, her menses appeared after six weeks' delay, and was on her last Monday night, when I saw her last. Yet she has had no further symptom of hystero-epilepsy, and this has been my sole treatment for two weeks, except I am using suppositories of iodoform in the vagina and holding or supporting the prolapsed uterus with cotton tampons.

I know very little about this chloride of gold, this being my first use of it, and I can't say that the benefit I have derived from its use is going to be permanent, as I have employed it only a fortnight. Yet, in that short time, it has accomplished wonders in my case of hystero-epilepsy.

